# Mutations in the SARS-CoV-2 spike RBD are responsible for stronger ACE2 binding and poor anti-SARS-CoV mAbs cross-neutralization

**DOI:** 10.1016/j.csbj.2020.11.002

**Published:** 2020-11-12

**Authors:** Masaud Shah, Bilal Ahmad, Sangdun Choi, Hyun Goo Woo

**Affiliations:** a Department of Physiology, Ajou University School of Medicine, Suwon, Republic of Korea; b Department of Molecular Science and Technology, Ajou University, Suwon, Republic of Korea

**Keywords:** COVID-19, mAb, SARS-CoV-2, Spike protein, Therapeutic peptides

## Abstract

Severe acute respiratory syndrome coronavirus 2 (SARS-CoV-2), which causes coronavirus disease 2019 (COVID-19), is a novel beta coronavirus. SARS-CoV-2 uses spike glycoprotein to interact with host angiotensin-converting enzyme 2 (ACE2) and ensure cell recognition. High infectivity of SARS-CoV-2 raises questions on spike-ACE2 binding affinity and its neutralization by anti-SARS-CoV monoclonal antibodies (mAbs). Here, we observed Val-to-Lys417 mutation in the receptor-binding domains (RBD) of SARS-CoV-2, which established a Lys-Asp electrostatic interaction enhancing its ACE2-binding. Pro-to-Ala475 substitution and Gly482 insertion in the **A**GSTPCN**G**V-loop of RBD possibly hinders neutralization of SARS-CoV-2 by anti-SARS-CoV mAbs. In addition, we identified unique and structurally conserved conformational-epitopes on RBDs, which can be potential therapeutic targets. Collectively, we provide new insights into the mechanisms underlying the high infectivity of SARS-CoV-2 and development of effective neutralizing agents.

## Graphical abstract

**Figure:**
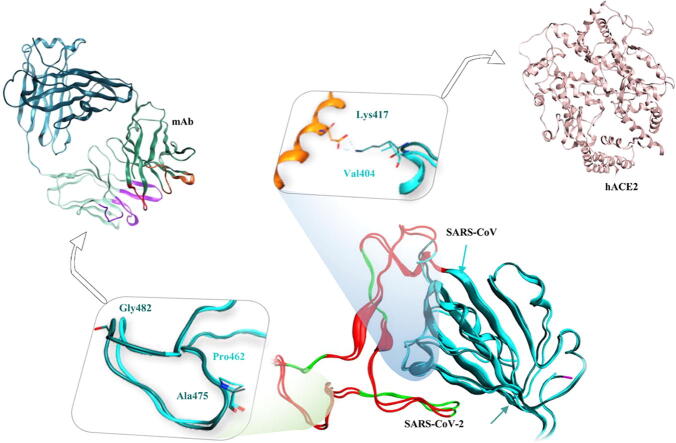

## 1 Introduction

SARS-CoV-2 causes coronavirus disease 2019 (COVID-19), which was initially reported in Wuhan city in China and disseminated around the world very quickly [1], [2]. However, the origin and global spatial spread of the virus, particularly in Europe and North America, has been hotly contested [3]. SARS-CoV-2 is a novel beta coronavirus [4], [5] and apparently transmits from human-to-human markedly faster than previously known coronaviruses, such as SARS-CoV and MERS-CoV [6], [7]. Although the epidemiological features of SARS-CoV-2 are largely unknown, asymptomatic transmission and poor self-quarantine measurements of infected persons are thought to be the most crucial reasons for the uncontrolled spread. In the current pandemic situation of COVID-19, there is an urgent need to develop effective therapeutics and vaccines. Several pre-existing anti-viral drugs and novel vaccines including Sputnik V, CoronavVac, mRNA-1273, d5-nCoV, inactivated vaccine, Covishield, and BNT162 are now on clinical trials [2], [8].

Among the mode of action mechanisms of the viral infection, cell-recognition and cell-entry are the most crucial steps that determine viral infectivity and pathogenesis [9]. Like SARS-CoV, SARS-CoV-2 uses spike (S) glycoprotein to interact with the human respiratory and epithelial cells expressing angiotensin-converting enzyme 2 (ACE2) receptors [10], [11]. The ectodomain of S protein is an ~1200 amino acid long class 1 fusion protein and normally exists in a trimeric metastable pre-fusion conformation of “laying or down”, which can undergo conformational rearrangement and acquire an ACE2-feasible conformation, i.e., “up or standing” [11], [12]. The “down” and “up” poses are differentiated due to the conformational rearrangement of the receptor binding domain (RBD, ~200 amino acid) in the S1 subunit of the S protein. The position switching of the RBD from down-to-up facilitates receptor binding while up-to-down helps the virus to evade the immune surveillance [12], [13]. The availability of receptor-binding determining region (RBDR) to the ACE2 is controlled by the hinge-like conformational motion of the RBD [11]. A mutation at the 614 amino acid i.e. Asp614-Gly has been reported to enhance the up conformation of the RBD that makes the virus more infectious and susceptible to neutralizing antibodies [14], [15]. Thus, S protein is indispensable for the virus survival and remains a priority target for antibodies and peptides-based biologics to curb viral entry. Although the receptor-binding mechanism of the spike protein has been widely studied and well-established now, we constructed an ectodomain model of the SARS-CoV-2 S protein in its pre-fusion form with respect to the two-conformation-states of RBD that pose a major challenge to the neutralizing antibodies and vaccines development. In addition, using the ACE2-RBD structures of SARS-CoV RBD (sRBD) and SARS-CoV-2 RBD (cRBD), we investigated their relative dynamic interaction, stability, and binding affinities. Using these structural-insights, we suggest peptides-based therapeutics that could possibly hinder the ACE2-RBD interaction. Further, we identified conformational epitopes on the cRBD that ought to be taken into account while investigating the cross-reactivity of anti-sRBD monoclonal antibodies (mAbs) or designing new mAbs and peptide therapeutics against SARS-CoV-2 S protein.

## 2 Methods

### 2.1 SARS-CoV-2 spike protein modeling

Almost all ectodomain trimeric structures of the SARS-CoV-2 spike protein that are mainly resolved through cryogenics electron microscopy (Cryo-EM) are incomplete, lacking vital structural information. This is because of poor electron density of the highly flexible looped regions that connect different motifs and subdomains in spike protein. Thus, understanding the structural insights of the trimeric spike requires extensive loop-modeling of the existing structures or new template-based modeling. As SARS-CoV and SARS-CoV-2 share significant identity in their spike proteins (>76% total identity; 92% in the S2 domain and 64% in the S1 domain) and by the time we conducted this study no ectodomain structures of the SARS-CoV-2 was reported; we used multiple SARS-CoV structures as template (PDB ID: 5X5B, 6ACG, and 5I08) to build the ectodomain (a.a 13–1122) spike protein model in Molecular Operating Environment (MOE) suit (2019.0102). The amino acid sequence of the spike protein that is among the first reported SARS-CoV-2 sequence and placed as reference sequence in NCBI was retrieved (accession #: NC_045512) and used in spike modeling. MOE modeling package uses a four-step procedure for model building i.e. initial partial geometry specification (to the templates), insertion and deletion (depending on the target and template sequence alignment), loop selection and sidechain packing (if template has missing loop structure), and final model selection and refinement (based on model scoring). Taking the RBD conformational position “up” or “down” into account, two trimeric spike models were generated. The spike-ACE2 complex was generated by structural superimposition of the modelled trimeric SARS-CoV-2 “up” and SARS-CoV structure, 6ACG. The models were further refined through the structural correction package and validated through the protein geometry (this package evaluate the model through Phi-Psi angles, dihedrals, rotamers energies etc.) package distributed in MOE. For the molecular dynamics simulation analysis, the ACE2-RBD regions were truncated from the trimeric spike-ACE2 complexes of both SARS-CoV-2 and SARS-CoV (PDB: 6ACG). Protein surface and patch analyses were performed in MOE as described previously [16]. Further details about the MOE-based modeling procedure and the protein-protein interface analysis have been described in our previous studies [17], [18], [19].

## 3 Spatial epitope prediction of mAbs docking onto SARS-CoV-2 RBD

For the mAbs, CR3014 and CR3022, modeling, an antibody modeling package of MOE was used and only the single chain variable fragments (scFv) were constructed [20]. The complementarity-determining regions (CDR) were annotated and numbered as described previously [21], [22]. Structural data of other mAbs including 80R, m396, F26G19 and s230 were obtained from PBD [23], [24], [25], [26]. For mAbs docking, a built-in protein–protein docking procedure was used in MOE suit. In docking simulation, CDR regions of mAbs were considered as ligand-sites instead of entire scFv regions.

Conformational epitopes of cRBD were predicted using Epipred implemented in SAbPred web server, utilizing the CDR information of an input mAb and predictsing conformational epitopes on a target protein [27], [28]. By calculating geometric fitting and knowledge-based asymmetrical antibody-antigen scoring, the epitopes of the cRBD were predicted and ranked on the basis of combined conformational matching of the antibody-antigen structures. The score of the epitope is calculated using the following formula:

$$\mathrm{Epitope}\;\mathrm{Score}=\sum_{n\in G}d\left(n\right)\mathrm{Pr}\left(T_{\mathrm{ab}},T_{\mathrm{ag}}\right)$$

where *T_ab_* and *T_ag_* are the amino acid types of the antibody and antigen residues, respectively, which belong to node *n*.

### 3.1 Molecular dynamics (MD) simulations

MD simulations were performed using GROMACS 2019.3. The RBD-ACE2 complexes were solvated with TIP3P water filled cubic box of dimension boundaries extended to 10 Å from protein atoms. To neutralize the charge of the simulation system the Na^+^/Cl^-^ counter ions were added and energy minimization was performed using CHARMM37 force field [29] and steepest descent algorithm. The energy-minimized systems were simulated for 0.2 ns with an NVT ensemble followed by 0.2 ns with NPT ensemble for equilibration, under constant temperature and pressure, respectively. The temperature and pressure were coupled with V-rescale and Parrinello-Rahman barostat methods [30], respectively. The long-range electrostatic interactions were computed using the particle mesh Ewald algorithm [31], while LINCS algorithm was applied to constrain the bond lengths [32]. After temperature and pressure equilibration, MD simulations were carried out for 30 ns for each system. A detailed procedure has been described in our previous studies [16], [18].

### 3.2 Binding free energy analysis

Since both the cRBD and sRBD bind to the same ACE2 protein, we used Molecular mechanics Poisson-Boltzmann surface area (MM-PBSA) approach [33] to calculate the relative binding free energies of both complexes. In GROMACS, the built-in tool g_mmpbsa and APBSA were called for the MMPBSA calculations. For g_mmpbsa analysis, the dielectric constant of the aqueous solvent was set to 80, and the interior dielectric constant was set to 4; the surface tension constant g was set to 0.022 kJ/mol. As g_mmpbsa tool is compatible with older versions of GROMACS (versions 5 or lower), the “tpr” files created by GROMACS 2019.3 were recreated through GROMACS 5.1 and used for binding energies calculations, as described previously [34]. The relative binding energies of the complexes were approximated according to the following energies terms.

$${\Delta G}_{\mathrm{bind}}={\Delta E}_{\mathrm{MM}}+{\Delta G}_{\mathrm{sol}}$$

Which is further extended as:

$${\Delta E}_{\mathrm{MM}}={\Delta E}_{\mathrm{cov}}+{\Delta E}_{\mathrm{elec}}+{\Delta E}_{\mathrm{vdW}}$$

where as;

$${\Delta E}_{\mathrm{cov}}={\Delta E}_{\mathrm{bond}}+{\Delta E}_{\mathrm{angle}}+{\Delta E}_{\mathrm{torsion}}$$

and;

$${\Delta G}_{\mathrm{sol}}={\Delta G}_{\mathrm{polar}}+{\Delta E}_{\mathrm{non}-\mathrm{polar}}$$

where ΔE_MM_ is gas-phase molecular mechanical energy change and ΔG_sol_ is the solvation free energy change upon binding. All these changes were computed via ensembles which are averaged over a set of conformations sampled throughout the simulation trajectories at 0.01 ns time interval. The ΔE_MM_ is calculated through molecular mechanics (MM) by combining three energy terms: the van der Waals energy change (ΔE_vdW_), the electrostatic energy change (ΔE_ele_), and the covalent energy change (ΔE_cov_) which further consists of change in the bond (ΔE_bond_), angle(ΔE_angle_) and torsion terms(ΔE_torsion_). The solvation free energy (ΔG_sol_) is computed by combining both polar and non-polar energies.

## 4 Results and discussion

### 4.1 Structural modeling of the SARS-CoV-2 spike and ACE2 interaction

**Fig. 1:**
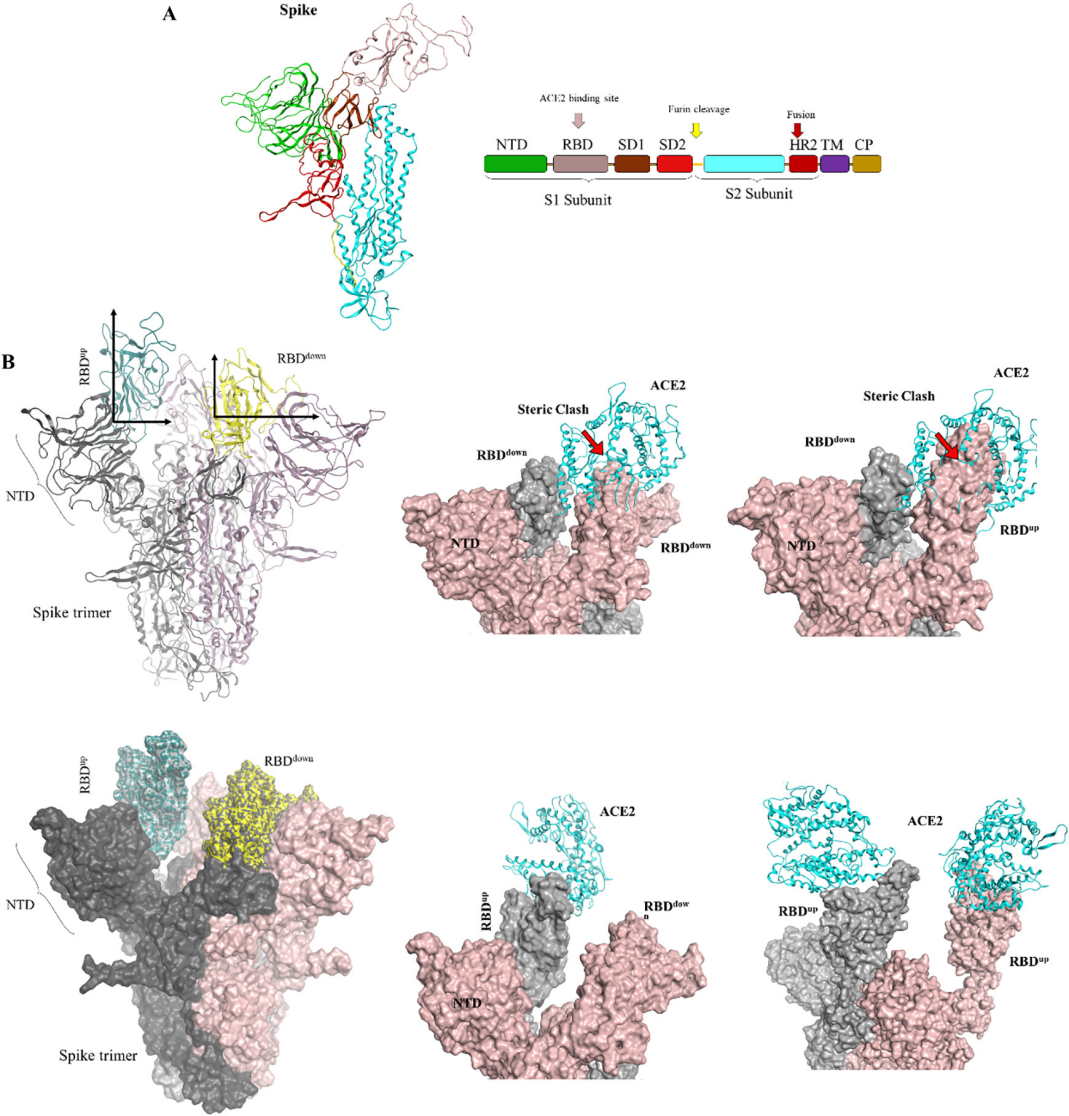
Modeling of the SARS-CoV-2 spike protein and its ACE2 binding mechanism. A) Domain architecture of the monomeric full-length S protein and the 3D monomeric spike model corresponding to a.a. 13–1122 in the ectodomain is shown. The furin-cleavage site at the junction of S1-S2 junction is highlighted with yellow arrow. B) The RBDs are displayed in receptor accessible “up” (cyan color) and non-accessible “down” (yellow color) conformation on the trimeric spike. ACE2 makes steric clash with the adjacent RBD if bound to RBD^down^. In RBD^up^ conformation, single trimeric spike can accommodate up to three ACE2 molecules, depending on the number of RBDs in up conformation. (For interpretation of the references to color in this figure legend, the reader is referred to the web version of this article.)

A full-length spike protein is composed of S1 and S2 subunits, which further contains sub-domains and motifs with distinct functions (Fig. 1A). Based on the hinge-like motion of the RBD of S1 subunit, the trimeric S protein exists in a transiently symmetric or asymmetric conformation. Recent studies revealed that the cRBD, like other coronaviruses, exhibit stochastic breathing-like movement, facilitating receptor binding to the exposed RBD and subsequent shedding of the S1 subunit [11], [12]. This hinge-like motion of the RBD is considered as one of the immune evasion strategy of the SARS-CoV-2 where the virus masks its RBD from host antibodies in down conformation and makes it available for the ACE2 in up position [12], [13]. Owing to the limitation in the availability of crucial structural information in the Cryo-EM SARS-CoV-2 spike protein structure (PDB ID: 6VSB, we modelled the monomeric and trimeric models of the SARS-CoV-2 S protein in different conformational states (with respect to the RBD position, and with or without ACE2) using SARS-CoV spike proteins as template. When compared with Cryo-EM structure, we could see a high degree similarity in the structural folds of both modeled protein and reported spike EM-structures in up and down conformations. To understand the structure more clearly and validate our model, the Cryo-EM structure and our models were superimposed in standing (in the presence of ACE2) and laying conformation. Total root mean square deviation (RMSD) of the backbone atoms of S protein in standing pose was 2.93 Å while that of the RBD region was 1.45 Å (Supplementary Fig. 1A). By contrast, RMSD values in the laying pose were 2.56 Å and 1.38 Å, respectively. This validation supports our model and further suggests that the structure modelled by Cryo-EM poorly capture the looped and highly flexible regions in spike protein, which is critical for the binding of ACE2 and antibodies. To investigate that the up conformation of RBD is indispensable for ACE2 binding, we constructed models where ACE2 was bound to both up and down RBD in trimeric spike. Considering the possibility of ACE2 binding to the down RBD where the RBD of the adjacent spike protomer may exist in up or down conformation, we observed that in both cases ACE2 may not bind to the down RBD due to the steric hindrance of the adjacent RBDs (Fig. 1B). On the contrary, ACE2 dock well onto the RBD in up conformation. One spike trimer could possibly accommodate two ACE2 proteins simultaneously, depending on the occurrence of two standing RBD at the same time. Depending on the state of ACE2, which exist in monomeric (solution form) as well as membrane-bound dimeric form [23], the one spike trimer–two ACE2 notion could be possible in two ways. First, two soluble ACE2 bind to two up RBDs on the same spike trimer (shown in Fig. 1B); second, two ACE2 in the membrane-bound dimeric form bind to two up RBD on two separate spike trimers (supplementary Fig. 1B). As a dimeric ACE2 is known to accommodate two RBD simultaneously [23], we suggest that soluble ACE2 may bind to the spike RBD more readily and stoichiometrically. This is because the binding of membrane-bound ACE2 is highly likely dependent on the spatial arrangement of the spike trimers on the virus surface, whereas soluble ACE2 can bind to any RBD available in up conformation. This is why soluble ACE2 or its derivative efficiently block the SARS-CoV-2 cell entry [35], [36].

**Fig. 2:**
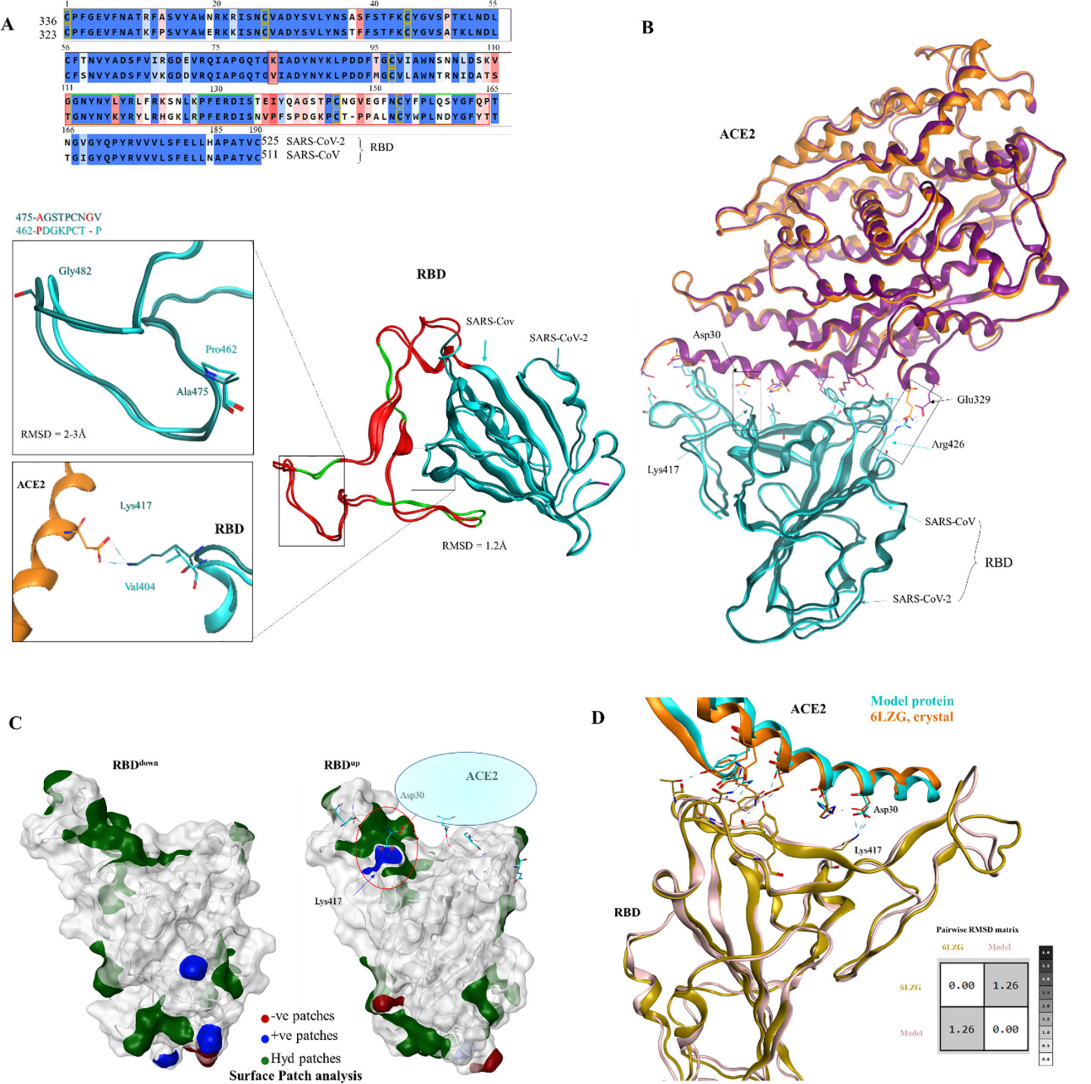
Comparative sequence and structural analysis of the SARS-CoV and SARS-CoV-2 RBD. A) RBD alignment (amino acid sequences) of the SARS-CoV-2 and SARS-CoV. Residue numbers at the start are according to the full-length S model and the top numbers represent RBD region only. The numbers at the start are used in RBD-ACE2 docking analysis, while the top numbers are used in RBD-mAbs docking analysis. The lower panel shows the superimposition of sRBD and cRBD. The enhanced box shows crucial mutations responsible for high receptor binding (Lys417) and antibody escape (Ala475, Gly482). B) The superimposed RBD-ACE2 complexes in both SARS-CoV and SARS-CoV-2 are shown, and the interface residues are depicted as sticks. Electrostatic contacts are highlighted in boxes. C) The surface patch analysis suggests the exposure of Lys417 in standing conformation (Asp30 belongs to ACE2 in the patch analysis). The color codes are displayed according to the charge of the patch. D) The SARS-CoV-2 RBD-ACE2 model is superimposed onto the reported complex resolved through X-ray crystallography. Their backbone RMSD values are displayed in the box.

Next, we sought to analyze and compare the interface cRBD-ACE2 with that of sRBD-ACE2. Sequence and structure comparison analyses revealed that RBDR of SARS-CoV-2 was substantially variable compared to that of SARS-CoV, which harbored few conserved motifs. The average RMSD for the whole cRBD and sRBD was ~ 1.1 Å, whereas the average RMSD for RBDR deviated by ~ 2–3 Å owing to the glycine insertion and other mutations (Fig. 2A). Both cRBD and sRBD established similar contacts with ACE2 residues due to the conserved motifs, although they are variable in RBDR region (Table 1). Interface analyses revealed that the electrostatic contact between Arg426 and Glu329 in sRBD-ACE2 was analogous to that of Lys417 and Asp30 contact in cRBD-ACE2 (Fig. 2B). However, this interaction was transient and break after the Asp30 of ACE2 established an intrachain contact with the nearby His34 (explained later). By performing protein patch analysis, we demonstrated that the standing cRBD exposes Lys417 that establishes strong electrostatic interaction with Asp30 of the ACE2, although this patch remains buried in the laying position of the cRBD (Fig. 2C). This finding indicates that the lysine 417 mutation in cRBD plays a crucial role in ACE2 recognition, which is otherwise substituted by hydrophobic valine in sRBD.

**Table 1:** Interface residues of cRBD and sRBD with ACE2 are shown at 0 ns and 20 ns of simulation. The loss of electrostatic contact can be noted in both states (bold residues).

RBD-ACE2 interface (0 ns)							RBD-ACE2 interface (20 ns)					
Virus	RBD	ACE2	Bond	E^kcal/mol^	Dist Å	Atom	RBD	ACE2	Bond	E^kcal/mol^	Dist Å	Atom
**SARS-CoV**	**Arg426**	**Glu329**	**IH**	**−35.88**	**2.99**	**–**	Tyr481	Lys353	H	−9.80	2.89	b-
	Gly482	Lys353	H	−7.20	2.80	b-	Tyr436	Asp38	H	−5.80	2.75	–
	Tyr491	Glu37	H	−4.90	2.56	–	Gly488	Gly354	H	−4.00	2.80	bb
	Tyr436	Asp38	H	−3.70	2.53	–	Thr486	Asn330	H	−3.40	2.83	b-
	Asp463	Ser19	I	−1.49	3.61	-b	Asp463	Gln24	H	−2.60	2.78	–
	Asn479	Glu35	H	−1.30	3.18	–	Thr486	Asp355	H	−1.70	2.60	–
	Thr486	Tyr41	H	−1.20	2.99	–	Tyr491	Gly354	A	−0.70	3.86	-b
	Asn473	Gln24	H	−1.00	2.89	–						
**SARS-CoV-2**	**Lys417**	**Asp30**	**IH**	**−30.83**	**2.71**	**–**	Gln493	Lys31	H	−11.60	2.85	–
	Tyr505	Glu37	H	−4.60	2.57	–	Tyr449	Asp38	H	−2.80	2.58	–
	Gly502	Lys353	H	−3.60	3.10	bb	Asn501	Lys353	H	−2.80	3.05	b-
	Gln498	Lys353	H	−2.90	3.00	–	Gln474	Gln24	H	−2.70	2.78	–
	Gln506	Gln325	H	−2.60	2.82	–	Asn487	Gln24	H	−2.40	2.85	–
	Asn487	Gln325	H	−1.60	2.80	–	Gln498	Lys353	H	−1.60	3.03	–
	Gln493	His34	H	−0.50	3.07	–	Thr500	Lys353	H	−1.40	2.71	b-
							Tyr489	Phe28	A	−0.60	3.75	b-
							Gln498	Tyr41	A	−0.60	4.52	–

VH, variable heavy chain; VL, variable light chains; H, hydrogen bond; A, aromatic bond; I, ionic (electrostatic) bond; bb, backbone atoms.

During the preparation of our manuscript, Yan R *et al*. demonstrated the interaction between SARS-CoV-2 RBD and ACE2 by performing Cryo-EM analysis [37], which is in line with our study supporting the reliability of our computational model. Another study has delineated the interface of ACE2 with a chimeric RBD through X-ray crystallography [38]. Overall, this chimeric structure shares structural folds with our model and recently reported Cryo-EM structure [37] and validates the cRBD-ACE2 interface residues in our model; nonetheless, the chimeric structure lacks the crucial mutation of Val-to-Lys417. This is because the core (scaffold) RBD in this structure was taken from SARS-CoV, which is considerably conserved between the two viruses with few mutations including Val-to-Lys417 (Supplementary Fig. 1C). As the high resolution crystal structure of the SARS-CoV-2 RBD with ACE2 has been reported during the course of this study, we validated our ACE2-RBD model by comparing the structure and interface with recently reported structure (6LZG) resolved through X-ray diffraction [39]. As expected, there was negligible differences in the overall RBDs (backbone atoms RMSD = 1.26 Å) structures and the ACE2-RBD interface residues were same in both complexes, including Lys417 (Fig. 2D). Therefore, we suggest that our computational model provides detailed understanding about the structural variation in cRBD and its interface with ACE2.

### 4.2 Mutation of Lys417 in cRBD may facilitate stronger interaction with ACE2

**Fig. 3:**
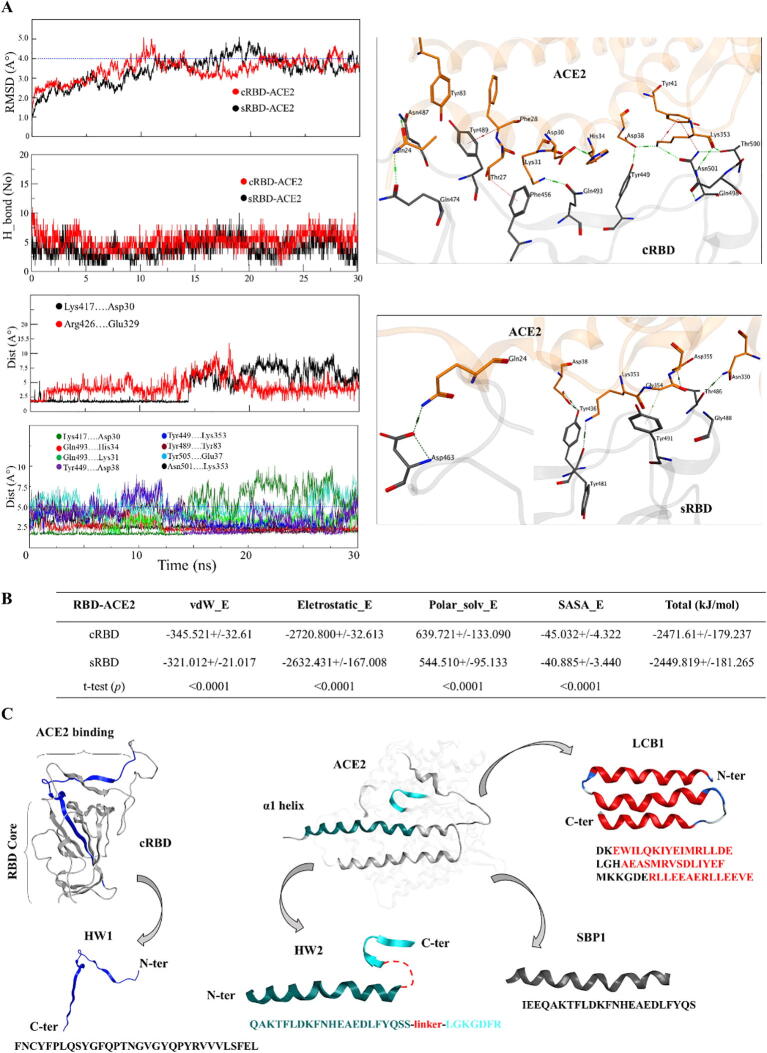
Binding affinity and interface analysis of the RBD-ACE2 complexes.A) The RMSD of the backbone atoms of the RBD-ACE2 complexes with reference to the 0 ns conformation are shown (top panel). Total number of hydrogen bonds between RBD-ACE2 interfaces is shown in the second to top panel. The minimum contact distances, as a function of time, between the oppositely charged and other important residues at the RBD-ACE2 interface are shown in the third and bottom panels, respectively. The interfaces (PDB frames extracted at 20 ns of simulation) of ACE2 with cRBD (top) and sRBD (bottom) are depicted. B) The binding free energies of the sRBD-ACE2 and cRBD-ACE2 are estimated through MM-PBSA and tabulated here. All energies were computed via ensembles, which are averaged over a set of conformations sampled throughout the simulation trajectory at 0.01 ns time interval. Statistical significance of the differential energies was calculated through *t*-test. The p-values (<0.05) indicate the significance of calculated energies and their differences between sRBD-ACE2 and cRBD-ACE2 complexes. C) Structure based cRBD-ACE2 blocking peptides designing. HW1 and HW2 peptides are suggested in this study and SBP1 and LCB1 are reported RBD-binding peptides.

Differing from SARS-CoV and SARS-related CoVs, the S protein of SARS-CoV-2 has furin cleavage site at the S1/S2 boundary as well as exhibits similar or more binding affinity towards ACE2, which might be responsible for the efficient spread of SARS-CoV-2 [40]. In addition to these two points, we next sought to identify mutations in cRBD that play critical roles in the stronger binding-tendency towards ACE2 as compared to sRBD. As the information is limited about the static conformation of a protein complex considering the changes of the binding interface in physiological condition, we simulated the complex structure of cRBD-ACE2 and compared this with the sRBD-ACE2 complex. The distances between interface residues were monitored as a function of time to trace the shifting, breaking, or formation of new bonds. Surface Plasmon resonance (SPR) and bio-layer interferometry (BLI) analyses have shown that cRBD-ACE2 interaction is stronger than sRBD-ACE2 interaction [11], [40], [41]. Supporting this, we also observed that the total number of hydrogen bonds remained similar throughout the simulation time in both sRBD-ACE2 and cRBD-ACE2 models (Fig. 3A). This result may imply that the stronger binding affinity of cRBD toward ACE2 might be attributed to the stronger interaction of Lys417-Asp30 compared to Arg426-Glu329. Interestingly, when the minimum interaction distances with respect to the simulation time was monitored, we observed that Lys417-Asp30 pair was more compact as compared to Arg426-Glu329 pair. Initially the residues in both pairs were ~1.4 Å apart; however, the Arg426-Glu329 pair separated by 2.6 Å, but the Lys417-Asp30 pair remained intact until the midpoint of the simulation. The bonds between both pairs broke at the same time point and remained separated by ~5 Å till the end of simulation (Fig. 3A). The relative strength and variation in the bond distance between these two pairs have been recently evaluated computationally, which were substantially in line with our findings [42]. These strong yet transient electrostatic contacts can partly explain the phenomena of receptor recognition and S1 shedding upon enzymatic cleavage of the S1-S2 junction. S protein transiently utilizes the RBD of S1 subunit for receptor recognition and sheds them during cell internalization. Thus, faster SARS-CoV-2 transmission as compared to SARS-CoV is, at least in part, might be facilitated by the robust Lys417-Asp30 interaction.

In addition, we observed that Tyr449, Tyr489, Gln493, and Asn501 in cRBD established strong hydrogen bonds with the interface residues of ACE2 and remained intact throughout the simulation (Fig. 3A). These results indicate that these residues are equally responsible for the relatively stronger interaction of cRBD with ACE2. To demonstrate our results more clearly, we captured the motions of these interface residues in animations (Supplementary movies 1 and 2), and calculated binding free energies for each complex along the simulation time. The vdW, electrostatic, and SASA energies of the cRBD-ACE2 were relatively stronger than that of sRBD-ACE2. Besides, the polar solvation energy of cRBD-ACE2 was relatively higher than sRBD-ACE2, which may compensate the differences in the other energies of these complexes, resulting in overall slightly higher total binding free energies for the cRBD-ACE2 (see Fig. 3B). These data are in agreement with recently reported study who estimated the binding-free energies of sRBD and cRBD-ACE2 complexes through AMBER tool [43]. Collectively, our structural modeling analyses could demonstrate stronger cRBD-ACE2 interaction compared to sRBD-ACE2 interaction. In addition, we demonstrated that Lys417 mutation may allow cRBD-ACE2 contact more readily, which may facilitate the rapid transmission of SARS-CoV-2 compared to SARS-CoV.

### 4.3 Druggability of the ACE2-RBD interface and decoy-based peptide inhibitors

With the help of structural information provided by the binding analysis of SARS-CoV-2 spike protein and ACE2, many groups have been actively involved in designing peptide antidotes that are able to block the receptor binding of the virus. A peptide, S_471–503_, derived from the ACE2 binding region of the sRBD has been able to hinder ACE2-sRBD interaction and thus SARS-CoV entry into the cell, as confirmed *in vitro* [44]. By comparing the cRBD region corresponding to the S_471–503_ (**ALNCYWPLNDYGFY**TTTGIGYQPYRVVVLSFEL) peptide, we found that the N-terminus (bold letters) of S_471–503_ and the corresponding cRBD region were considerably different; however, the C-terminus portion (non-bold letters) was 100% identical to cRBD (see Fig. 2A, **aligned sequences**). Owing to the difference in the N-terminal half, S_471–503_ may not hinder SARS-CoV-2 cell entry as it exhibited in the SARS-CoV related study. We suggest that a peptide HW1, FNC**YFPLQSYGFQPTNGVGYQ**PYRVVVLSFEL may abolish the interaction between cRBD and ACE2. However, the c-terminal region, PYRVVVLSFEL, of HW1 is predominantly hydrophobic and belongs to the core region of the RBD (Fig. 3C). Overall, this peptide is physiologically unstable and may aggregate, losing its’ target specificity in solution.

Alternatively, we observed that the helical region of the ACE2 remains stable during simulation that establish strong electrostatic and hydrophobic contacts with the RBDR of both SARS-CoV and SARS-CoV-2 (Table 1, Supplementary movies 1 & 2), probably holding the potency to bind RBD. In fact, the interface between sRBD and ACE2 has been exploited in the past and ACE2-derived peptides have been used to block the SARS-CoV cell entry. A peptide, P6 (EEQAKTFLDKFNHEAEDLFYQSS-G-LG**K**GDFR), constructed by the glycine linkage of two separate segments of ACE2 has been able to exhibit efficient antiviral activity (IC_50_ = 0.1 µM) [45]. Others and we found that cRBD and sRBD interact with the overall same helical peptide (α1 helix) of the ACE2 with some differing interface residues from the RBDs (see Table 1). Particularly, the two glutamic acids, EE, at the N-terminus of ACE2 did not make stable contacts with both s- and cRBDs and were exposed to the solvent. Lysine 353 (bold letter in P6) is crucial for RBD binding, establishing multiple stable hydrogen bonds with both s- and cRBDs (Fig. 3A, Table 1). Thus, we suggest that a peptide HW2, QAKTFLDKFNHEAEDLFYQSS-linker-LGKGDFR (Fig. 3C) may hold the optimum capacity to engage RBD and halt its binding to ACE2. More recently, a short peptide SBP1derived from the α1 helix of ACE2 was shown to bind SARS-CoV-2 RBD that was expressed in insect; however, the results were not reproduced with human and other insect-derived RBDs [46]. This finding suggests that either the α1 helix of ACE2 is not sufficient to bind RBD or it loses helicity and RBD-binding ability in solution state. A new study has shown that stabilizing the helical fold of α1 helix (LCB1 & AHB1-2, Fig. 3C) retains its RBD-binding capacity and effectively inhibit the SARS-CoV-2 cell entry [47]. Conclusively, we suggest that merely α1 helix derived peptides, in the absence of structural constrains or LGKGDFR motif, may poorly bind RBD and hinder its binding to ACE2.

### 4.4 Identification of epitopes on cRBD that bind to SARS-CoV-2 mAbs

**Fig. 4:**
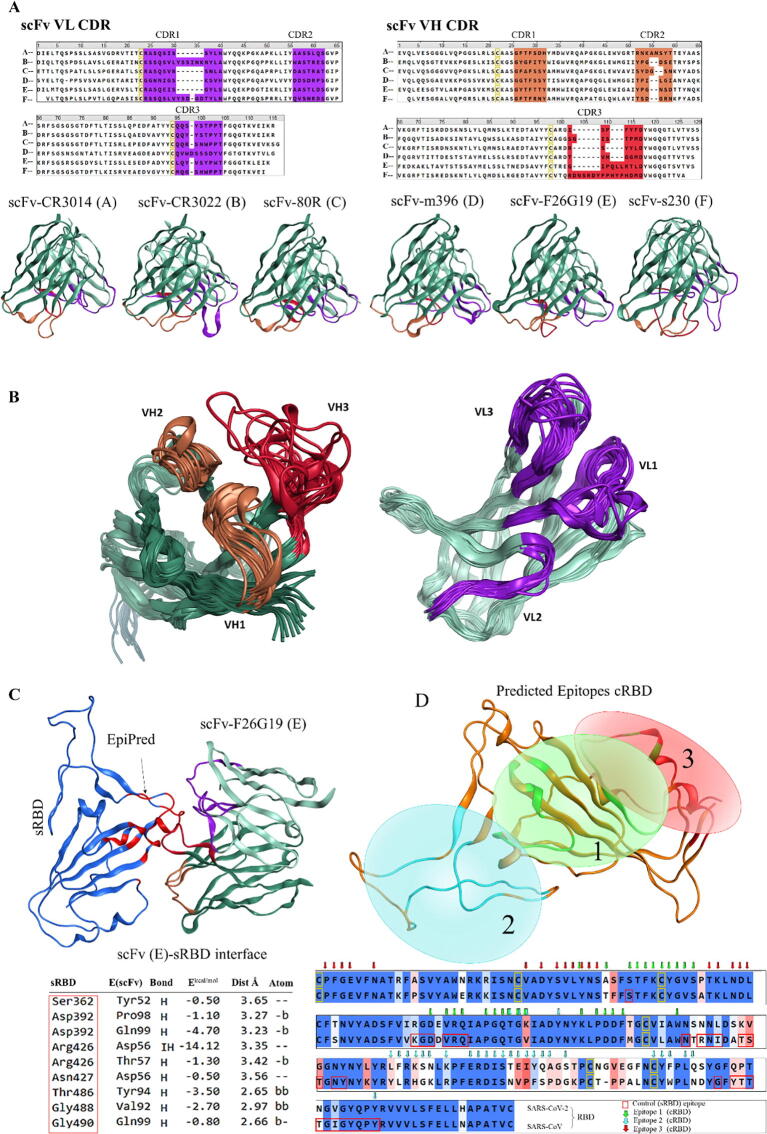
Epitope mapping of the cRBD and complementarity-determining region (CDR) annotation of the mAbs. A) Anti-sRBD mAbs (single-chain variable fragments (scFv)) and their CDRs are shown. B) The variable light (VL) and variable heavy (VH) chains of the scFv regions of the reported anti-SARS-CoV-2 RBD mAbs are superimposed and the CDR regions are annotated according to Chothia and Lesk numbering scheme. C) The epitope prediction was validated through sRBD-F26G19 complex (PDB ID: 3BGF). The tabular interface is reported in the crystal structure while red boxes in the aligned sequences show the EpiPred predicted epitope. D) Conformational epitopes predicted with reference to six known anti-sRBD mAbs are highlighted and encircled. Residues participating in epitopes are indicated with arrows in the aligned cRBD and sRBD a.a. sequences (the arrow colors correspond to their respective epitopes). (For interpretation of the references to color in this figure legend, the reader is referred to the web version of this article.)

SARS-CoV-2 and SARS-CoV belong to the genus *betacoronavisus* of the family coronaviridae and share considerable sequences in the RBD region of S protein [6], [40], which allow researchers to delineate the cross-reactivity of anti-sRBD mAbs with cRBD [11], [41]. To investigate whether the previously known SARS-CoV mAbs can bind cRBD, we explored the binding potential of the cRBD with the SARS-CoV mAbs including 80R [23], m396 [25], F26G19 [24], s230 [26], CR3014, and CR3022 [48]. The structures of mAbs were obtained from PDB [for 80R [23], m396 [25], F26G19 [24], and s230 [26]] or modeled them [for CR3014 and CR3022 [30]] (for details see Methods). The variable heavy (VH) and variable light (VL) chains of scFv regions in these mAbs were aligned and their CDRs were annotated. These models revealed that the VL-CDR1 of CR3022 and s230 were relatively longer and more similar as compared to the VL-CDR1 of the other mAbs; in addition, the VH-CDR3 of s230 was more expanded than those of the other mAbs (Fig. 4A). Differences in the sequence and length of the CDRs indicate that these mAbs recognize distinct epitopes on the RBD and may not overlap thoroughly. Over a short period, more than two dozen of SARS-CoV-2 S protein neutralizing mAbs have been identified and structurally elucidated. We compared the sequence and structures of CDR regions of these mAbs with that of CR3022 and F26G19. We found that the immunoglobulin G heavy-chain variable region 3 (in other words the VH-CDR3) and to some extent the VL-CDR1 in these mAbs are diversified and utilized to target the RBD of spike protein (Fig. 4B, Supplementary Tables 1 & 2).

Next we sought to predict conformational epitopes of cRBD using structural information of the mAbs. To ensure the authenticity of the epitope prediction, the co-crystal structure of sRBD-F26G19 was used as control. We observed that epitope 1 completely overlapped with the experimental result, supporting the reliability of our analysis (Fig. 4C). Among the predicted cRBD epitopes, the residues in epitope 2 were mainly composed with highly variable regions between sRBD and cRBD (cyan color arrows in the aligned sequences). In contrast, the residues of the epitope 1 and 3 were significantly conserved between sRBD and cRBD (epitope 1, 93%; epitope 3, 100%, Fig. 4D). This result implies that the anti-SARS-CoV sRBD mAbs recognizing epitope 1 or epitope 3 could bind the cRBD and may hinder its receptor binding. However, the epitope 2 region was highly variable between cRBD and sRBD, therefore the anti-sRBD mAbs recognizing epitope 2 may not be able to bind or neutralize cRBD.

### 4.5 Highly conserved epitopes of cRBD are promising target for anti-SARS-CoV-2 agents

**Fig. 5:**
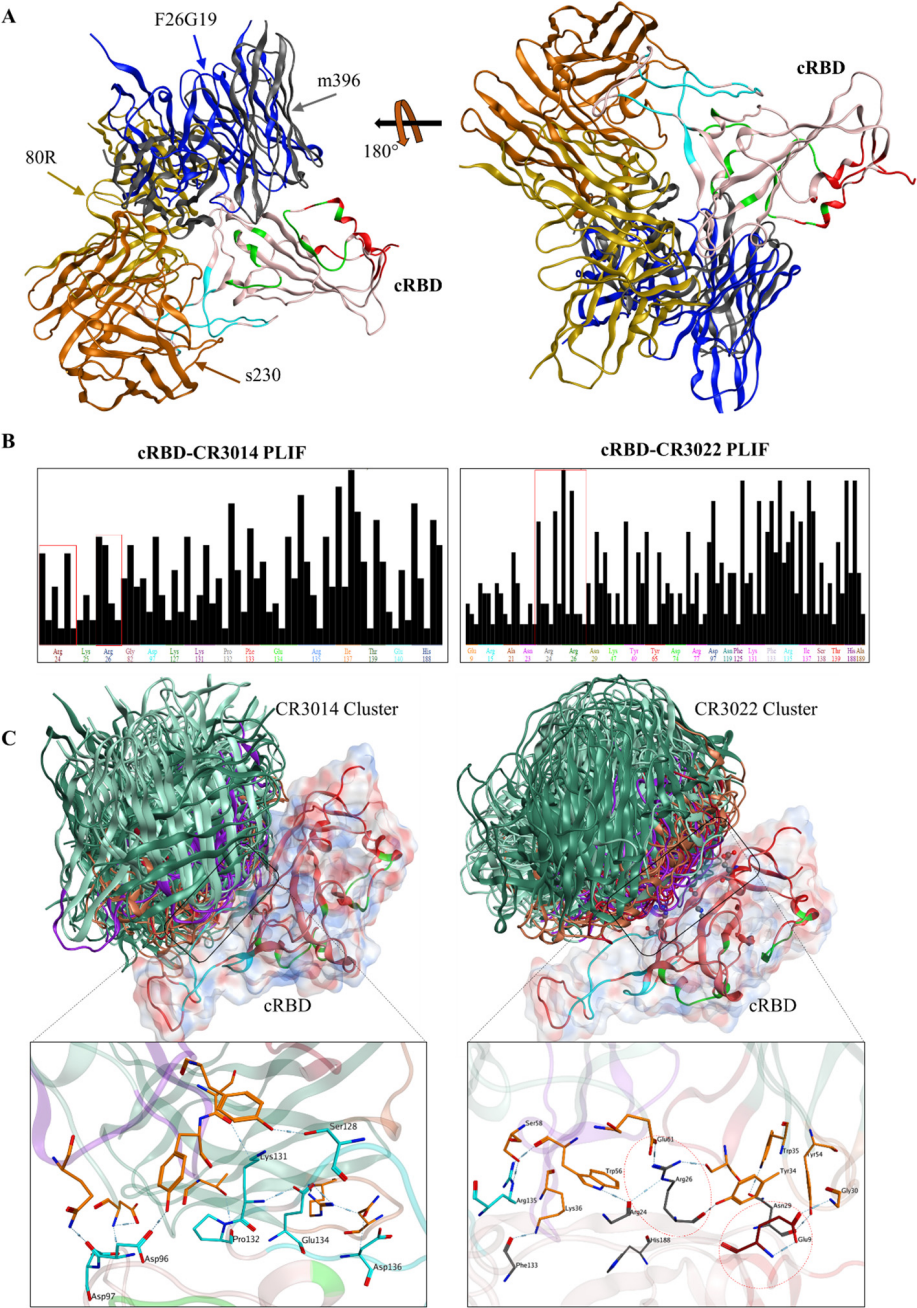
RBD-mAbs interface and their protein ligand interaction fingerprints (PLIF) analysis. A) The binding interface of SARS-CoV mAbs are displayed with respect to the predicted epitopes on cRBD (Green = Epitope 1, Cyan = Epitope 2, Red = Epitope 3; explained in Fig. 4). B) PLIF analysis of the CR3014 and CR3022 mAbs with cRBD. C) CR3014 cluster around epitope 2, which is highly variable between sRBD (*left*) and cRBD (*right*). CR3022 cluster near epitope 3, which is conserved between cRBD and sRBD. (For interpretation of the references to color in this figure legend, the reader is referred to the web version of this article.)

Recent studies comprising SPR and BLI analyses have demonstrated that the sRBD mAbs including m396, 80R, s230, and CR3014 are not able to recognize cRBD [11], [41], although the reason for failure was not understood. To evaluate the reason, we placed or docked the scFv regions of these sRBD mAbs onto cRBD revealing their interface residues (Table 2). s230 and 80R interacted with a part of the overlapping residues at the hypervariable RBDR region (epitope 2) of cRBD; this could possibly explain their negative binding in the previous SPR and BLI experiments [11], [41]. m396 and F26G19 were partly overlapped onto the residues at non-epitope regions (Fig. 5A), suggesting that these mAbs may not bind cRBD. The binding affinity of F26G19 with cRBD has not been studied yet requiring further evaluation in future. Taken together, we suggest that these m396, 80R, s230, and F26G19 mAbs recognize non-conserved or non-epitope regions of cRBD, and therefore might not be able to block the cRBD interaction with ACE2. Interestingly, cRBD escapes from the anti-sRBD mAbs even though cRBD can bind to ACE2 with high affinity. This could be partly explained by the structural differences of binding regions between them. Anti-sRBD mAbs have CDR that are very specific and recognize conformational epitopes on RBD, while ACE2 utilize a long helix that binds longitudinally to RBD (Table 3).

**Table 2:** Iinterface residues of cRBD with anti-SARS mAbs.

mAB Type		cRBD	scFv	Bond	E^kcal/mol^	Dist Å	Atom
**m396**	**VH**	Thr169	Ser31	H	−2.00	2.73	b-
		Gly171	Thr33	H	−2.30	2.94	-b
		Asp74	Asn58	H	−3.10	2.77	–
		Gln175	Val97	H	−4.00	2.92	b-
	**VL**	Thr45	Ser93	H	−1.70	2.76	–
		Arg77	Ser94	H	−1.10	3.21	b-
		Arg77	Asp95	IH	−19.24	2.89	–
**F26G19**	**VH**	Ser44	Thr31	H	−1.50	2.80	-b
		Ser44	Tyr52	H	−1.60	2.93	–
		Asn106	Tyr52	A	−1.20	4.05	–
		Asn106	Asn54	H	−4.20	2.74	-b
		Asn108	Asp56	H	−6.70	2.99	–
		Asn109	Asp56	H	−6.00	2.97	–
		Val172	Gln95	H	−0.80	3.02	-b
	**VL**	Gly171	Val92	H	−1.00	3.11	bb
		Thr169	Tyr94	H	−5.10	2.88	bb
**80R**	**VH**	Gly165	Ser101	H	−1.70	2.81	b-
		Tyr174	Arg100	H	−0.50	3.12	–
	**VL**	Val114	Trp226	A	−0.50	4.51	–
		Asn119	Arg162	H	−11.70	2.83	–
		Gly154	Thr206	H	−3.20	2.74	b-
		Cys157	Ser197	H	−3.00	2.70	b-
		Gln162	Thr185	H	−2.10	2.77	–
**s230**	**VH**	Gln162	Arg54	H	−2.70	2.87	–
		Phe125	Asn55	A	−0.80	4.61	–
		Asn156	Asp60	H	−3.30	2.82	–
		Cys157	Lys63	H	−2.80	3.58	–
		Leu124	Tyr104	H	−3.10	2.62	-b
		Tyr90	Tyr104	A	−0.00	3.91	–

VH, variable heavy chain; VL, variable light chains; H, hydrogen bond; A, aromatic bond; I, ionic (electrostatic) bond; bb, backbone atoms. Residue numbers of the cRBD should be tallied with the top numbers in Fig. 2A.

**Table 3:** Interface residues of cRBD with docked CR3014 and CR3022 mAbs.

**mAB Type**		**cRBD**	**scFv**	**Bond**	**E**^kcal/mol^	**Dist Å**	**Atom**
**CR3014**	**VL**	Asp96	Gln27	H	−2.00	3.21	b-
		Asp96	Tyr92	H	−2.40	2.62	–
		Asp97	Asp1	IH	−13.963	2.80	-b
		Ser128	Tyr32	H	−2.00	3.74	–
		Lys131	Ser91	H	−0.600	3.28	-b
		Pro132	Thr94	H	−1.60	3.01	b-
	**VH**	Glu134	Arg54	IH	−26.192	2.78	–
		Asp136	Asn58	H	−0.50	3.48	b-
		His188	Arg71	H	−0.60	3.27	b-
**CR3022**	**VL**	Arg26	Glu61	IH	−14.70	3.26	–
		Arg135	Tyr56	H	−8.00	2.75	b-
		Arg135	Ser58	H	−3.40	2.96	–
		Phe133	Lys36	H	−2.90	2.87	-b
		Arg24	Tyr56	H	−2.60	3.24	-b
	**VH**	Glu9	Gly30	H	−6.80	2.80	b-
		Arg26	Ser102	H	−3.80	2.88	–
		Arg26	Tyr34	H	−2.50	2.66	-b
		Leu4	Thr33	H	−2.30	2.78	-b
		Asn29	Trp54	H	−2.90	2.61	-b

VH, variable heavy chain; VL, variable light chains; H, hydrogen bond; A, aromatic bond; I, Ionic (electrostatic) bond; bb, backbone atoms. Residue numbers of the cRBD should be tallied with the top numbers in Fig. 2A.

In addition, we observed a mutation at Ala475 in cRBD, which corresponded to Pro462 in sRBD (see Fig. 1A). A previous study has shown that CR3014 mAb was not effective on the mutant Pro462Leu viruses, although it could prevent lung damage and SARS-CoV shedding in ferrets [48]. We found a glycine insertion mutation in the same loop (475-**A**GSTPCN**G**V-483), lengthening the loop RMSD to 2–3 Å (see Fig. 1C). This might be the reason why the previous BLI study could not demonstrate the binding of cRBD with CR3014 [41]. With this concern, we performed epitope mapping and protein ligand interaction fingerprints (PLIF) analyses to further evaluate the binding of CR3014. CR3014 was clustered around the same **A**GSTPCN**G**V-loop, a part of epitope 2, implying that the epitope 2-targeting sRBD mAbs may not be able to bind cRBD (Fig. 5B). Likewise, the sRBD mAbs recognizing the epitopes in variable RBDR regions may also not be able to bind cRBD. Thus, we suggest that mAbs or therapeutic peptides that bind to a conserved epitope on RBD may hinder the interaction of both SARS-CoV and SARS-CoV-2 spike with ACE2.

CR3022 has been reported to completely neutralize the CR3014 escape SARS-CoV mutants (i.e., Pro462Leu) and synergize the neutralizing effect of CR3014 without competing with its epitopes [48]. A recent study has also demonstrated that CR3022 does not compete with ACE2-binidng site of cRBD and exhibit binding to cRBD in the BLI analysis; in contrast, other mAbs, such as CR3014, m396, and MERS-CoV neutralizing mAb m336 were not able to bind to cRBD [41]. These results imply that CR3022 but not the other mAbs bind the conserved epitope of RBD in SARS-CoV-2 and SARS-CoV. To evaluate this, we performed an antibody docking procedure and calculated PLIF based on 100 docked poses of the CR3022-cRBD complex. Differing from CR3014, CR3022 was clustered over Arg24 and Arg26 and interacted with Glu19, which we designated as epitope 3 in the cRBD (Fig. 5B, C). Recently, Park *et al*. have performed computational analyses to demonstrate whether the previously known anti-MERS-CoV and anti-SARS-CoV mAbs can bind and neutralize cRBD [49]. However, this study did not consider the fact that the spike protein, particularly the RBD of SARS-CoV and MERS-CoV, have significant variations, which may preclude neutralization of cRBD by anti-SARS-CoV and anti-MERS-CoV mAbs [50]. In their docking analysis, the binding of CR3022 and s230 overlapped on the same interface of cRBD. In contrast, we and Tian *et al.* [41] have demonstrated that there is no overlap between CR3022 and the ACE2-binding region of cRBD (see Supplementary Fig. 2A). A crystal structure analysis also revealed that s230 bind to the ACE2-binding region of sRBD [26]. To validate these results, we superimposed the structures of s230-sRBD and ACE2-sRBD complexes, which revealed that ACE2 and s230 were overlapped with the same interface of sRBD (Supplementary Fig. 2B). We also found that CR3022 did not compete with the ACE2 and CR3014 interfaces of cRBD, which was consistent with the results of previous studies for SARS-CoV [48] and the recent SARS-CoV-2 [41] (Supplementary Fig. 2A). Notably, we found that CR3022 recognized a highly conserved region partly overlapping with epitope 3 (Fig. 5C).

**Fig. 6:**
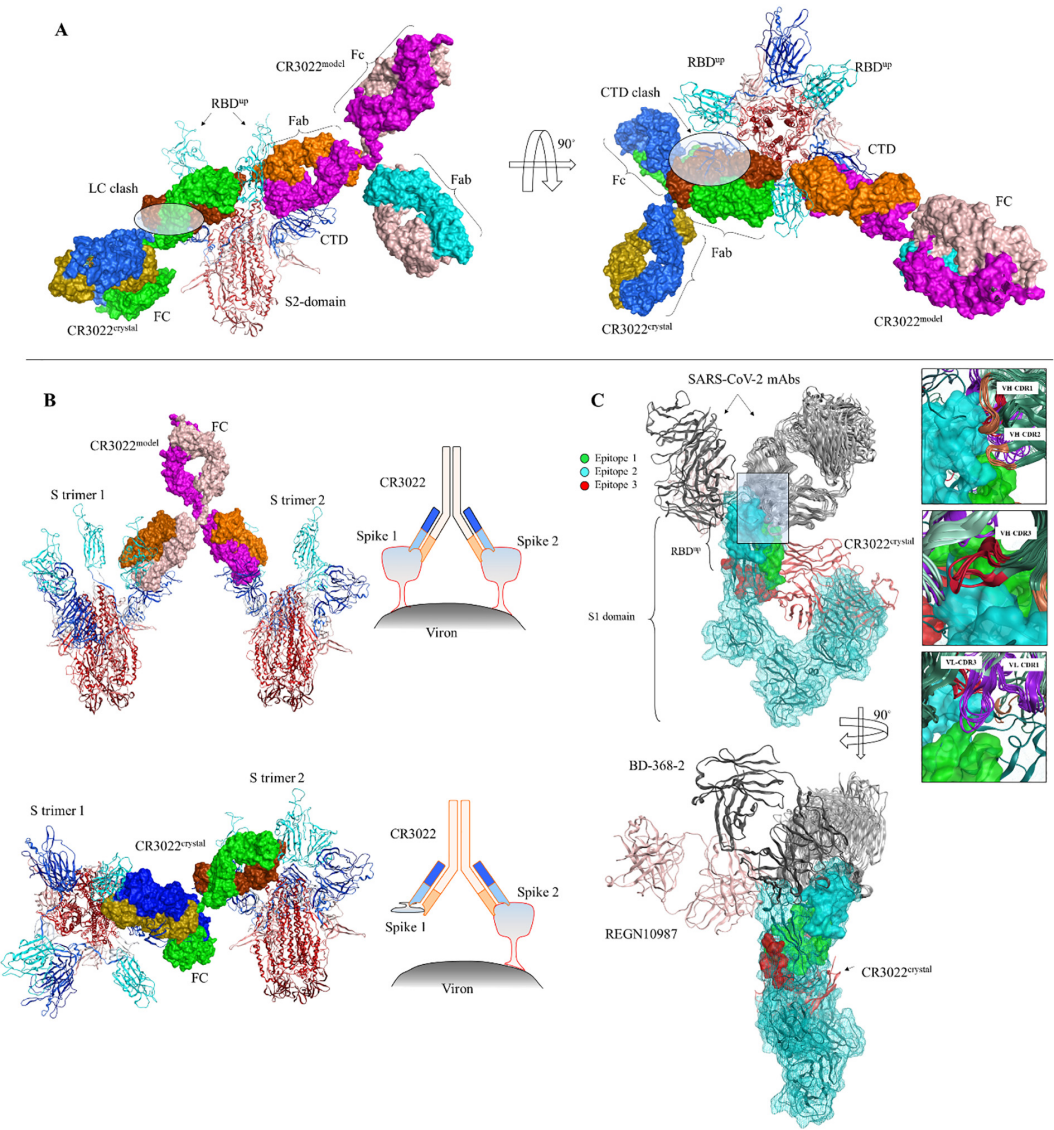
The binding mechanism of mAbs to SARS-CoV-2 spike RBD. A) CR3022 IgG is bound to trimeric SARS-CoV-2 spike in two conformational states. One conformation is suggested in our study (CR3022^model^) and the other conformation (CR3022^crystal^) is based on experimentally reported information. The highlighted area in the ovals indicate the clash between light chain constant (LC) region of the CR3022^crystal^ and C-terminal domain (CTD) of the adjacent S protomer. B) According to the model suggested in this study, CR3022^model^ can bind to two RBDs in nearby spikes attached to the viron surface, whereas CR3022^crystal^ cannot make such contacts due to the unfavorable spatial arrangement of the two spike attached to one CR3022^crystal^. C) Nineteen anti-SARS-CoV-2 mAbs and CR3022 are superimposed onto single S1 subunit of the SARS-CoV-2 spike and the epitopes are highlighted on the RBD region.

During the preparation of this manuscript, a new study has shown the binding of CR3022 with cRBD through X-ray diffraction analysis, suggesting that the CR3022 Fab binds to a cryptic epitope (epitope 3 in our study) on the cRBD with substantially lower affinity as compared to sRBD [51]. Full-length CR3022 IgG also exhibited similar binding affinity towards both cRBD and sRBD, but could not neutralize SARS-CoV-2. The difference in the binding affinities of CR3022 Fab and IgG with cRBD and its inability to neutralize SARS-CoV-2 needs further investigation. We further speculated the binding mechanism of CR3022 IgG with SARS-CoV-2 trimeric spike. Yuan et al. suggested that CR3022 could bind to a trimeric spike in two or three RBD up states, but not single up conformation due to steric hindrance. We observed that the light chain constant region of CR3022 in their suggested model clash with CTD of adjacent or same spike protomer, regardless of the RBD up position (Fig. 6A). Conversely, we suggest that epitope 3 can accommodate the Fab and CR3022 IgG differently without any clash with the surrounding protomers. We further suggest that a single CR3022 can bind to two RBDs of two different protomers in nearby trimers and an RBD^up^ can be assessed by the Fab regardless of the up or down conformation of the adjacent RBD (Fig. 6B). Further investigations including site directed mutagenesis and binding analysis to the trimeric spike are required to confirm the authenticity of these models.

Since dozens of SARS-CoV-2 spike-neutralizing mAbs have been reported now, we wished to investigate their epitopes on the cRBD. Nineteen mAbs bound to the cRBD were obtained from PDB database and their CDRs were compared with those of sRBD binding mAbs. Most of the SARS-CoV-2 spike neutralizing antibodies, with some exceptions, were bound to the RBDR region of cRBD (Fig. 6C). Surprisingly, all three CDRs in the VH region of these mAbs make contacts with the epitope 2. Whereas, the CDR3 in VL was docked into the epitope 1. This suggests that the RBDR region in cRBD is highly immunogenic due to the differences in the RBDR regions of SARS-CoV and SARS-CoV-2, producing highly specific and distinct antibodies in the body to neutralize their spike proteins. This could possibly explain the reason why anti-SARS-CoV mAbs are not effective against SARS-CoV-2. The cRBD binding mAbs can hinder its interaction with ACE2 even if they minimally bind or do not bind to the RBDR region. In fact, some mAbs can simultaneously bind to cRBD at distinct epitopes and abrogate its biding to ACE2 [52]. Hansen et al. generated nine cRBD-bindng mAbs from genetically humanized mice and COVID-19 convalescent patients and identified their epitopes on cRBD through hydrogendeuterium exchange mass spectrometry (HDX-MS). We found that the epitopes suggested by HDX-MS were non-linear and partly or fully overlapped with the three epitopes suggested in our study [52]. One mAb, REGN10987, which does not compete with ACE2, could simultaneously bind cRBD in the presence of REGN10933, which recognize epitope 2 (Fig. 6C). Similar antibody cocktail strategy has also been reported by other group, where two RBD-neutralizing antibody bind two non-overlapping epitopes on cRBD [53]. This study suggests that BD-629 binds to the RBDR (epitope 2), and BD-368-2 binds to a non-overlapping epitope (epitope not suggested in our study) on the opposite side of epitope 2 (Fig. 6C). Overall, these findings suggest that the RBD of SARS-CoV-2 is highly immunogenic and harbor multiple but overlapping epitopes.

In summary, we suggest that Lys417 mutation in cRBD acquires stronger electrostatic interaction with ACE2, which may facilitate faster receptor-recognition of cells. This interaction is further strengthened by electrostatic and hydrophobic contacts at the cRBD-ACE2 interface. In addition, we identified a conserved epitope on RBD, which might be the target for developing new peptide therapeutics or mAb for neutralizing SARS-CoV-2. Undoubtedly, our findings provide new insights into the underlying mechanisms of the high infectivity of SARS-CoV-2, which may be helpful for developing new effective neutralizing agents against SARS-CoV-2.

## Declaration of Competing Interest

The authors declare that they have no known competing financial interests or personal relationships that could have appeared to influence the work reported in this paper.
