# Supplementary material for: Mutations in the SARS-CoV-2 spike RBD are responsible for stronger ACE2 binding and poor anti-SARS-CoV mAbs cross-neutralization
Source: Comput Struct Biotechnol J. 2020 Nov 12;18:3402–14. doi: 10.1016/j.csbj.2020.11.002 (PMC7657873; doi:10.1016/j.csbj.2020.11.002)
Supplement: Supplementary data 1 [file mmc1.docx]

**Mutations in the SARS-CoV-2 Spike RBD are responsible for stronger ACE2 binding and poor anti-SARS-CoV mAbs cross-neutralization**

Masaud Shah^a^, Bilal Ahmad^b^, Sangdun Choi^b^, Hyun Goo Woo ^a^*

1. Department of Physiology, Ajou University School of Medicine, Suwon, Republic of Korea
2. Department of Molecular Science and Technology, Ajou University, Suwon, Republic of Korea

***Corresponding author:** Hyun Goo Woo, M.D., Ph.D., Department of Physiology, Ajou University School of Medicine, 164 Worldcup-ro, Yeongtong-gu, Suwon, Korea; Tel: 82-31-219-5045, Fax number: 82-31-219-5049, E-mail address: [hg@ajou.ac.kr](mailto:hg@ajou.ac.kr).

**Supplementary Data.**

**Supplementary Table 1.** Light chain variable (VL) region CDRs of the anti-SARS-CoV-2 mAbs are selected (according to the Chothia and LesK numbering) from the PDB structure. Anti-SARS-CoV mAbs are highlighted in bold.

| **PDB ID** | **CDR VL1** | **CDR VL2** | **CDR VL3** |
| --- | --- | --- | --- |
| 6XC3 (**CR3022)** | KSSQSVLYSSINKNYLA | WASTRES | QQYYSTPYT |
| 3BGF (**F26G19**) | RASQEISGYLS | AASTLDS | LQYVSYPWT |
| 6XDG | QASQDITNYLN | AASNLET | QQYDNLPLT |
| 6XDG | TGTSSDVGGYNYVS | DVSKRPS | NSLTSISTWV |
| 7CHC | RASQGVSSFLA | GASSRAT | QQYGSSPRT |
| 7CHC | RSSQSLLHSNGYNYLD | LGSNRAS | MQALQTPGT |
| 6XC2 | RASQGISSYLA | AASTLQS | QQLNSYPPKFT |
| 6XC7 | RASQSVSSYLA | GASSRAT | QQYGSSPRT |
| 6XE1 | RASQSVSSSYLA | GASSRAT | QQYGSSPQT |
| 7C01 | RASQSISRYLN | AASSLQS | QQSYSTPPEYT |
| 7BYR | RASQSISSWLA | KASSLES | QQYNSYPYT |
| 7JX3 | RASQTVSSTSLA | GASSRAT | QQHDTSLT |
| 7JX3 | TRSSGSIASNYVQ | EDNQRPS | QSYDSSNQV |
| 7JX3 | RASQSIGSYLN | AASSLQS | QQSYVSPTYT |
| 7CHF | RASQGISSDLA | AASTLQS | QQLNSDLYT |
| 7JMP | TGTSSDVGSYNLVS | EVTKRPS | CSYAGSSTWV |
| 7JXD | TGSSGSIASNYVQ | EDNQRPS | QSYDSSNHVV |
| 7JMW | QASQDISNYLN | DASNLET | QQYDNPPLT |
| 6XKQ | TGTSSDLGAYHFVT | GVRKRPS | SSYAGNNDFV |
| 7CAI | RASQSISSNLH | YASQSIS | QQTNFWPYI |
| 7JV6 | GSSTGAVTSGHYPY | DTSNKHS | LLSYSGARGV |
| 6XCN | TGTSSDVGGYKYVS | EVSKRPS | SSYEGSNNFVV |

**Supplementary Table 2.** Heavy chain variable (VH) region CDRs of the anti-SARS-CoV-2 mAbs are selected (according to the Chothia and LesK numbering) from the PDB structure. Anti-SARS-CoV mAbs are highlighted in bold.

| **PDB ID** | **CDR VH1** | **CDR VH2** | **CDR VH3** |
| --- | --- | --- | --- |
| 6XC3 (**CR3022**) | GYGFITY | YPGDSET | SGISTPMD |
| 3BGF (**F26G19**) | GYTFTTY | YPGNSDT | GIPQLLRTLD |
| 6XDG | GFTFSDY | TYSGSTI | RGTTMVPFD |
| 6XDG | GFTFSNY | SYDGSNK | SDYGDYLLV |
| 7CHC | EFIVSRN | YSGGST | YGDYYFD |
| 7CHC | GFAFTTY | SDGGGSA | RGRGLYDYVWGSKD |
| 6XC2 | LTVSSN | YSGGST | LDVYGLD |
| 6XC3 | GLTVSSN | YSGGST | LDVYGLD |
| 6XC7 | GFTVSSN | YSGGST | FGDFYFD |
| 6XE1 | GVIVSSN | YSGGST | LDVSGGMD |
| 7K43 | GYTFTGY | NPISSGT | APFYDFWSGYSYFD |
| 7BYR | GYTFTSY | NTNTGNP | QGGSSWYRDYYYGMD |
| 7JX3 | GYPFTSY | STYNGNT | YTRGAWFGESLIGGFD |
| 7JX3 | GFTFSNA | KSKTDGGT | SETYYYDSSGPFD |
| 7JX3 | GFTFSSY | GTAGDT | DSSGYYYYFD |
| 7K4N | GFTFTSS | VVGSGNT | YCSGGSCSDGFD |
| 7CHF | GIIVSSN | YSGGST | LGPYGMD |
| 7CHB | GITVSSN | YSGGST | LGEAGGMD |
| 6XKQ | GYNFTNF | SGYNGDT | DNYGFPYNGMD |
| 7CAI | GYSFSNY | DPFNGGT | EYDPYYVMD |
| 7JW0 | FTFSSYD | GTAGDT | DSSGYYYYFD |

**Supplementary Figure 1.**

**A)** The homology model and recently reported cryo-EM structures of full-length Spike (PDB ID: 6VSB) and RBD in standing and laying poses are superimposed. The missing regions (ACE2 binding loops) in cryo-EM are shown. **B**) The binding of two membrane-bound dimeric ACE2 to one trimeric spike with two RBD^up^ conformations. **C**) The chimeric crystal structure of the SARS-CoV-2 RBD in complex with ACE2 (PDB ID: 6VW1) is superimposed on the recently reported Cryo-EM (PDB ID: 6M17) and our modelled cRBD-ACE2 complexes. The cyan color in the chimeric crystal structure corresponds to the SARS-CoV RBD scaffold and the blue part belongs to SARS-CoV-2 RBD. The Lys-to-Val substitution in cRBD with respect to its interaction with Asp30 of ACE2 is highlighted in the enhanced box.

**
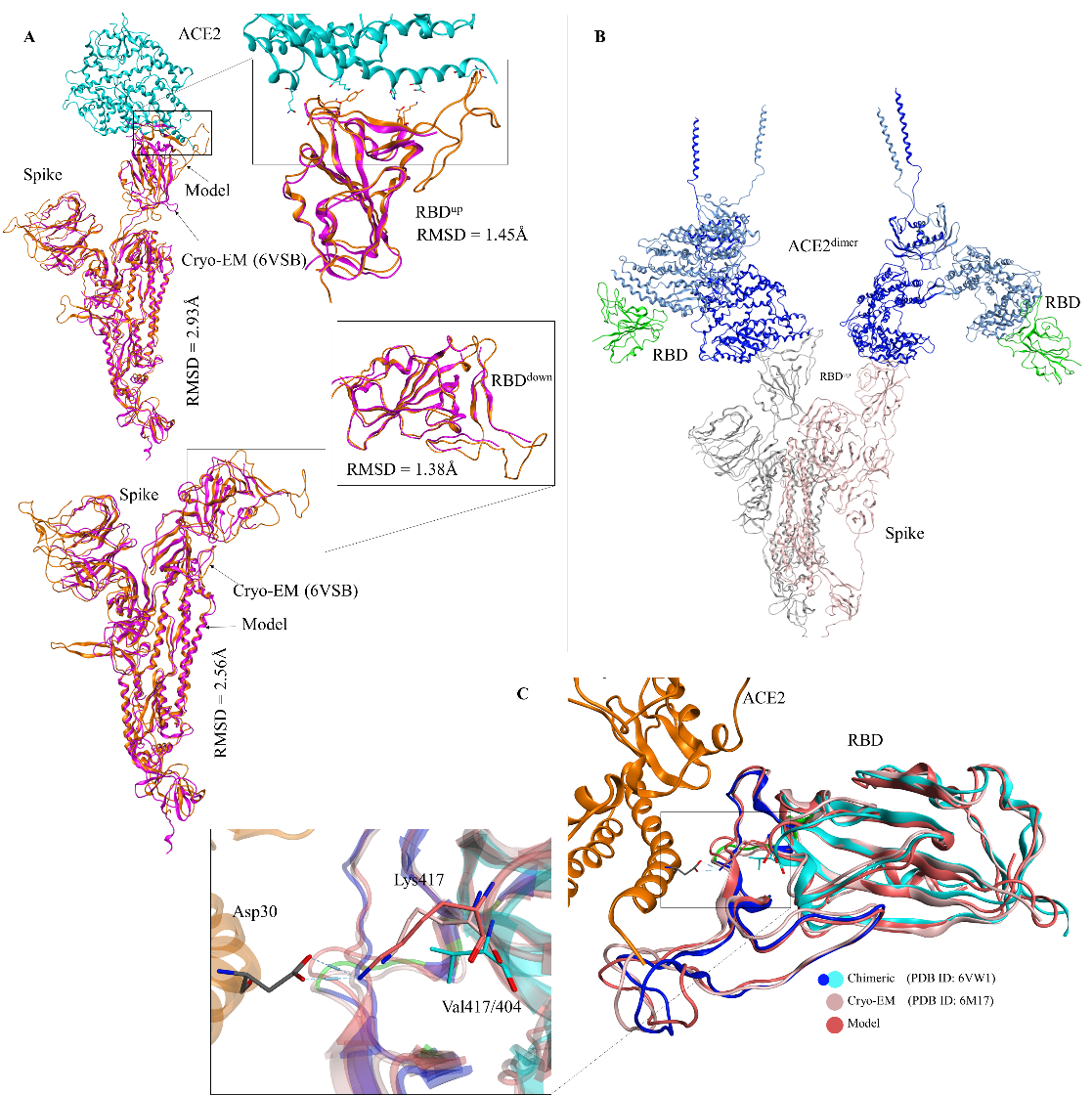
**

**Supplementary Figure 2.**

**A)** CR3022 does not overlap with the ACE2 and CR3014 binding interface of cRBD. **B)** The overlapping interface of the anti-sRBD mAB, S230, with ACE2 is shown. This superimposed model suggests that the variable heavy (VH) chain of S230 completely overlap with the ACE2 binding interface of sRBD. This model was generated by superimposing the 6NB7 and 6ACG structures retrieved from the protein data bank (PDB)**.**

**
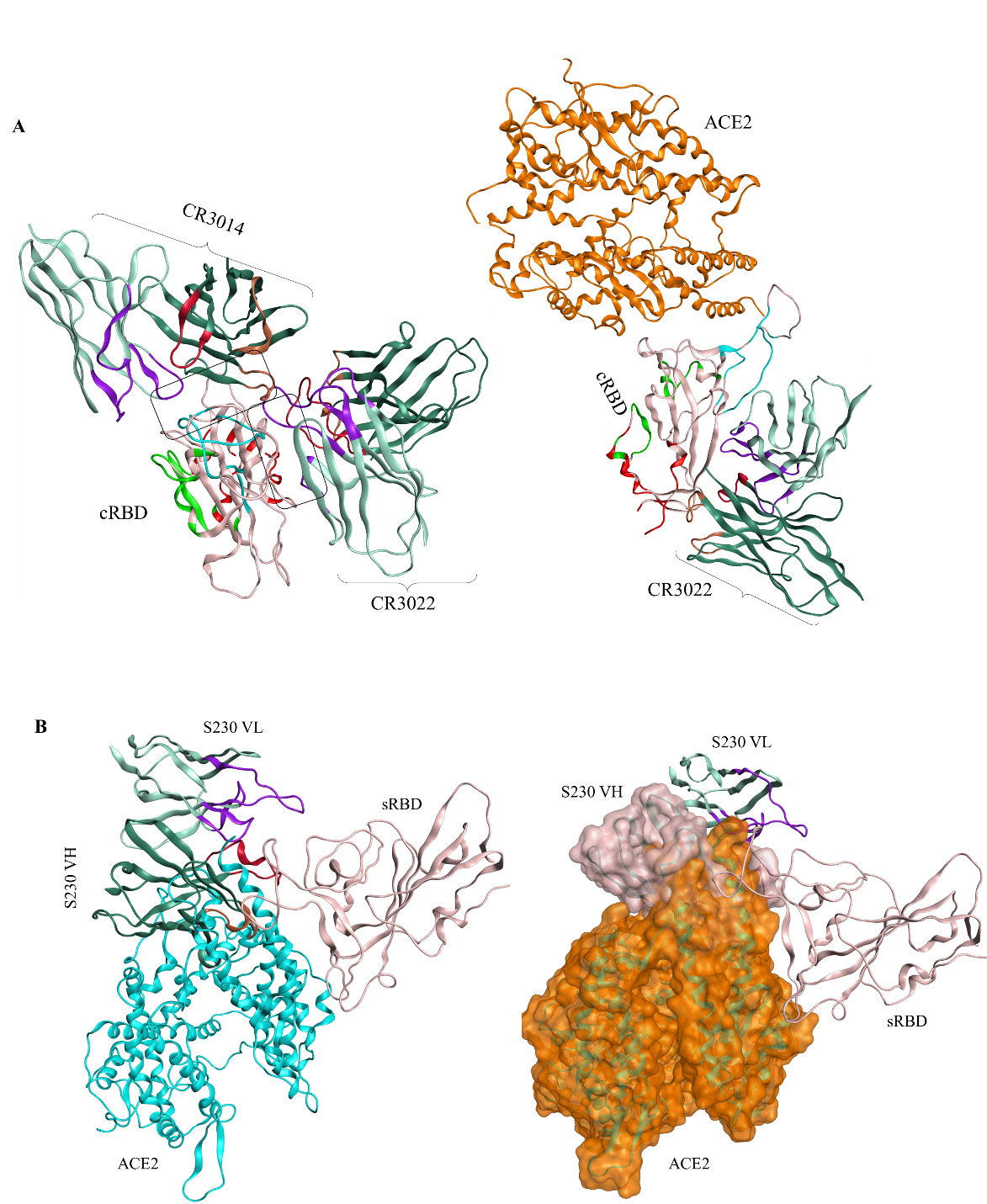
**

**Supplementary movie 1.** This animation is based on the structural coordinates extracted from the molecular dynamic trajectory. The cyan colored cartoon represents ACE2 and the brown colored cartoon represents SARS-CoV-2 cRBD. The interacting residues are depicted and labelled accordingly. The electrostatic contact between Lys417 and Asp30 can be seen.

**Supplementary movie 2.** This animation is based on the structural coordinates extracted from the molecular dynamic trajectory. The purple colored cartoon represents ACE2 and the brown colored cartoon represents SARS-CoV sRBD. The interacting residues are depicted and labelled accordingly. The electrostatic contact between Arg426 and Glu329 can be seen.
